# Pancreatic adenocarcinoma treated with surgical resection, toceranib phosphate and firocoxib in a dog: a case report

**DOI:** 10.1007/s11259-024-10349-5

**Published:** 2024-03-08

**Authors:** Manuel Fuertes-Recuero, Esther Vázquez-Fernández, Guillermo Lizasoain-Sánz, Amanda Arroba-Alonso, Alejandro Sánchez-López, Elena Martínez-de-Merlo, María Suárez-Redondo, Gustavo Ortiz-Diez

**Affiliations:** 1 Department of Physiology, College of Veterinary Medicine, Complutense University of Madrid, Avda. Puerta de Hierro s/n, Madrid, 28040 Spain; 2 Veterinary Teaching Hospital, Complutense University of Madrid, Avda. Puerta de Hierro s/n, Madrid, 28040 Spain; 3 VISAVET Health Surveillance Centre, Complutense University of Madrid, Madrid, 28040 Spain; 4 Department of Animal Medicine and Surgery, College of Veterinary Medicine, Complutense University of Madrid, Avda. Puerta de Hierro s/n, Madrid, 28040 Spain

**Keywords:** Pancreatic adenocarcinoma, Dog, Surgical resection and toceranib phosphate

## Abstract

Exocrine pancreatic carcinomas are rarely reported in dogs. A ductal pancreatic adenocarcinoma in a 10-year-old intact beagle is described in this report. The diagnosis was made based on clinical signs, imaging (abdominal ultrasound and CT scan) and histopathology. Treatment consisted of partial right lobe pancreatectomy followed by adjuvant therapy with toceranib phosphate (Palladia®) and firocoxib (Previcox®) for six months. The treatment was well tolerated, and the survival time was 445 days. To our knowledge, this is the longest survival reported in the literature for a dog diagnosed with exocrine pancreatic adenocarcinoma. The results described here may contribute to provide a better understanding about this neoplasia and potential treatment options.

## Introduction

Canine pancreatic adenocarcinomas are uncommon aggressive tumors arising from the exocrine pancreas. They can be divided into those arising from pancreatic acinar cells and those arising from the pancreatic ductular epithelium. Neoplasms of the exocrine pancreas are rarely reported in companion animals (< 0.5% of all cancers) (Withrow et al. 2020).

The common clinical signs associated with pancreatic carcinomas are nonspecific, such as anorexia, vomiting, diarrhea and abdominal pain (Aupperle-Lellbach et al. 2019). Blood count abnormalities usually include an inflammatory leukogram, and abnormalities in serum biochemistry include elevated alkaline phosphatase, alanine aminotransferase, amylase and lipase (Pinard et al. 2021; Musser and Johannes 2021). Metastases are often present prior to the diagnosis, which often leads to the patient’s euthanasia in a short period of time. A retrospective study in 23 dogs described that metastatic disease was detected in 78% of cases at the time of diagnosis, and the median survival time prior to necropsy was 1 day (range 0–51 days), with an overall survival time of 8 days (Pinard et al. 2021). These factors could contribute to the current scarcity of literature in veterinary medicine with only a few studies in cats (Linderman et al. 2013; Todd and Nguyen 2020; Rosario et al. 2023) and even fewer in dogs (Bennett et al. 2001; Pinard et al. 2021; Musser and Johannes 2021). In contrast, this neoplasia is widely described in human medicine, where treatment is mainly based on surgery, chemotherapy, radiotherapy and/or targeted therapy, depending on the stage of the disease and the resectability of the primary mass (Thompson and Wood 2020). Surgery stands out as the primary choice in canine patients, as radiotherapy and chemotherapy have shown limited value (Withrow et al. 2020; Pinard et al. 2021).

This report describes the case of a dog, that was diagnosed with pancreatic adenocarcinoma, which was surgically removed and treated with toceranib phosphate and firocoxib. Complete remission of clinical signs was achieved, and the patient had a progression-free interval (PFI) of 305 days, and 445 days of survival.

## Case presentation

**Fig. 1:**
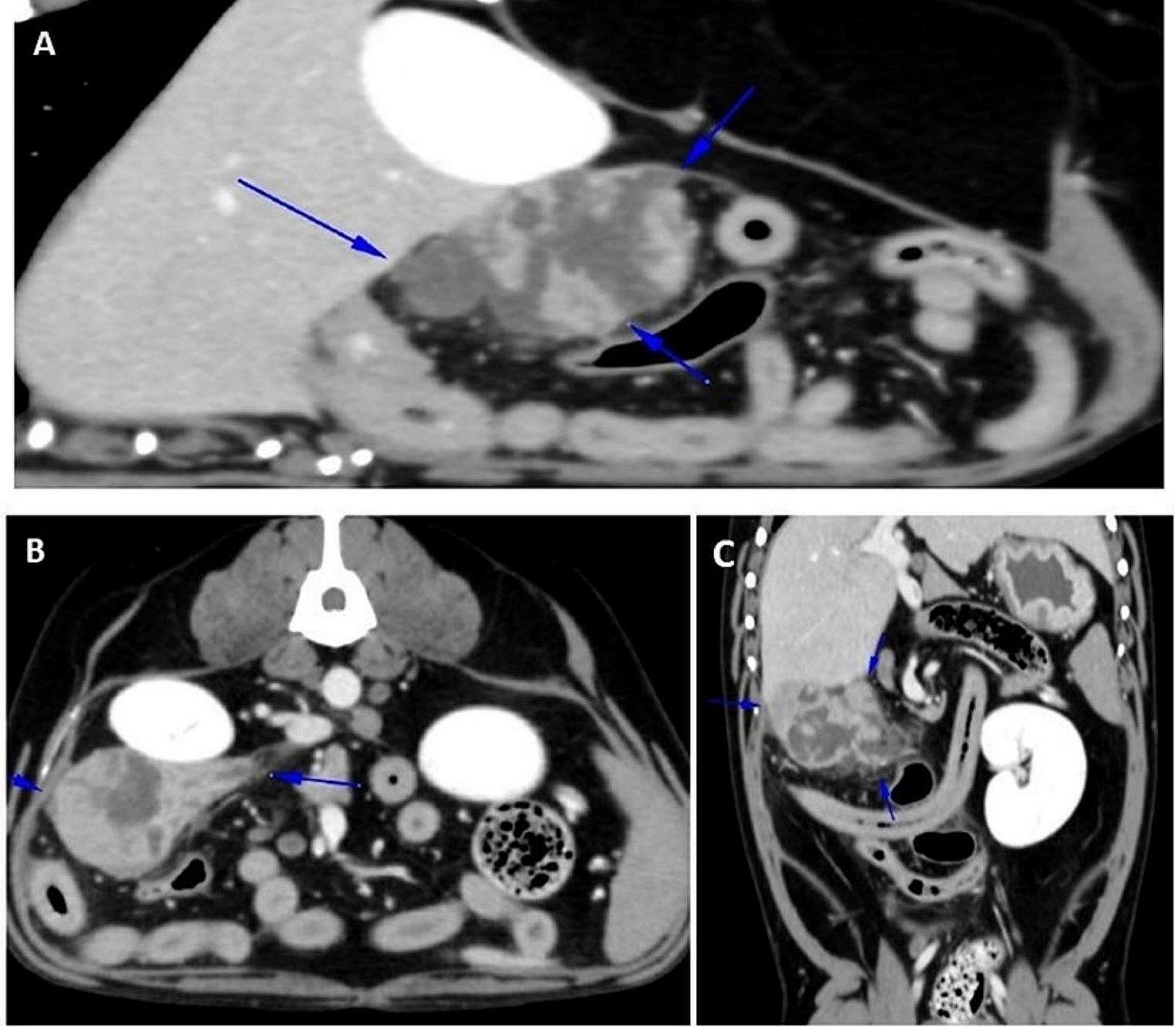
Abdominal CT postcontrast images showing a heterogeneous mass(blue arrows) in the cranial abdomen in the distal area of the right pancreatic lobe, adjacent to the right hepatic lobes, the ipsilateral kidney, and the ascending colon. (a) Sagittal plane. (b) Axial plane. (c) Coronal plane

A 10-year-old intact male Beagle dog was referred with anorexia, vomiting, abdominal pain and lethargy of two weeks duration, with no other recent history of other illness. On physical examination, moderate pain was noted in the cranial abdomen. No abnormal parameters were found in the blood count and serum biochemistry performed. Abdominal ultrasound (Canon Aplio i700) was performed, in which a 6 cm mass was detected in the right cranial abdomen. Based on the ultrasound findings, a thoracic and abdominal CT scan (Toshiba Aquilion 64-slice) with the administration of intravenous contrast (Optiray® 320 mg/ml, 1.8 ml/kg) was performed to assess the extent of the lesion and to investigate possible metastatic lesions. The abdominal CT scan showed a large mass (6 cm in length x 4.1 cm in width and 3.5 cm in height) in the cranial abdomen with a pancreatic origin. The mass involved the distal aspect of the right pancreatic lobe, was heterogeneous and had multiple hypoattenuated areas within it. No metastatic lesions were observed on the thoracic and abdominal CT scan. Differential diagnoses included neoplasm or, less likely, other types of masses, such as a granuloma, abscess or other (Fig. 1).

A percutaneous ultrasound-guided aspirate was performed. Cytology showed inflammatory changes of pancreatic parenchyma, which were compatible with neutrophilic inflammation, although neoplasia could not be ruled out. The patient’s abdominal pain became more severe after the tests, and required hospitalisation and symptomatic treatment with methadone (0,2 mg/kg/6 h IV; Semfortan®10 mg/mL), maropitant (1 mg/kg/24 h IV: Cerenia® 10 mg/ml) and intravenous fluid therapy. The patient was discharged after 24 h with ambulatory treatment. After 4 days, signs had not improved, and the patient was again admitted to the hospital. A naso-oesophageal tube was placed to facilitate assisted feeding, and the next day an exploratory laparotomy was performed.

A firm mass, surrounded by omentum, measuring 6 cm in length x 4.1 cm in width and 3.5 cm in height, was confirmed in the right lobe of the pancreas, at the site described on the ultrasound and CT scan. A partial pancreatectomy of the distal right lobe was performed, with a 2 cm margin of macroscopically healthy pancreatic tissue, using a combination of blunt dissection, vessel ligation, and bipolar hemostasis. No abnormalities were found in the liver or other abdominal organs consistent with metastasis, and no enlargement of the abdominal lymph nodes was observed. The dissection of the mass did not affect the accessory pancreatic duct. The rest of the right lobe and the left lobe appeared normal. The excised pancreatic tissue was submitted for histological evaluation and bacteriological culture. The latter turned out to be negative. During the same anesthesia, the nasal feeding tube was replaced by an oesophageal tube.

Recovery was uneventful. After surgery, significant clinical improvement was noted. The patient became more active, started to eat without assistance and was discharged 3 days post-surgery, after removing the oesophageal tube. Postoperative medications included tramadol, cefalexine and metronidazole.

**Fig. 2:**
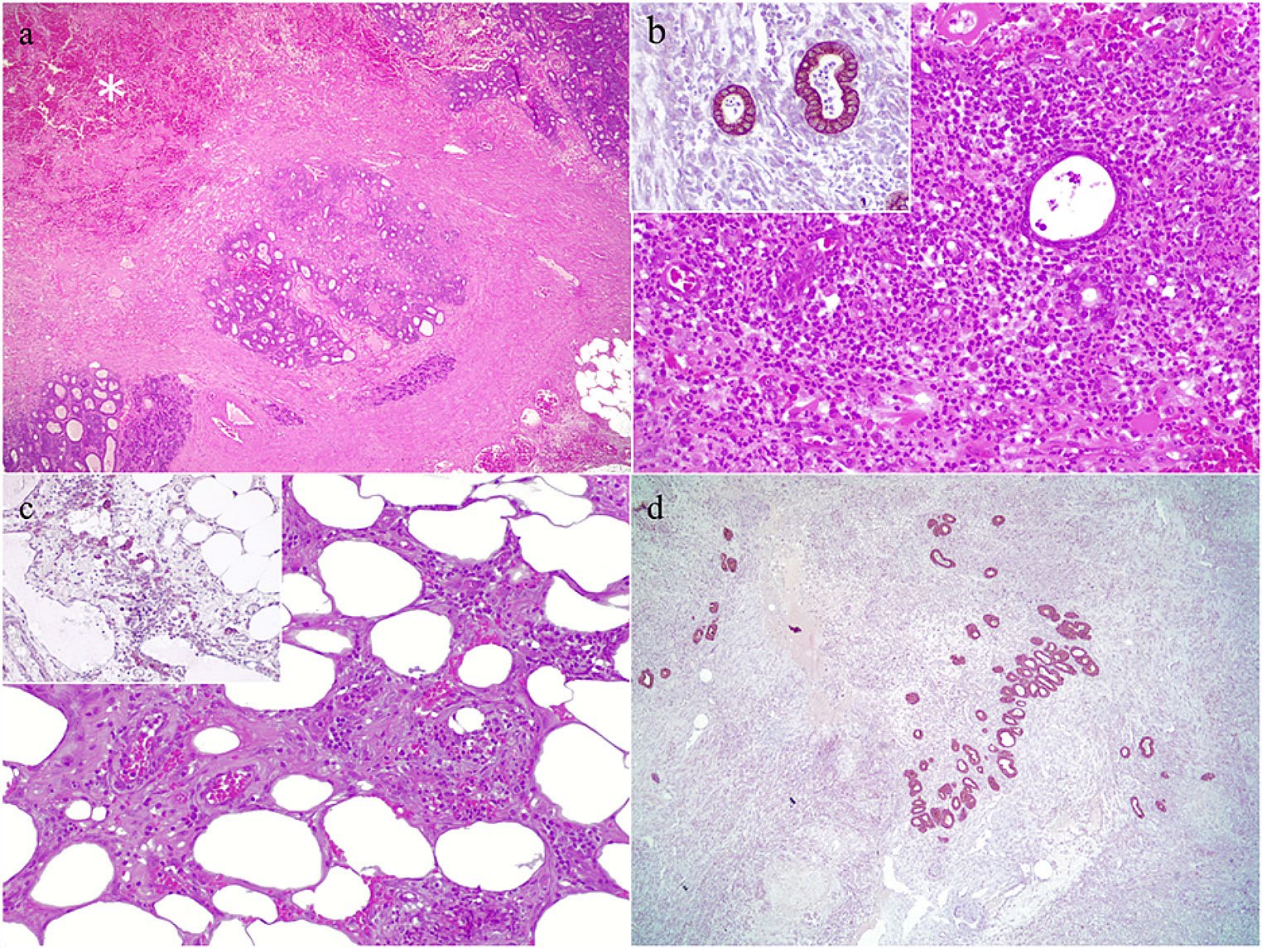
(a) Pancreas. Ductal adenocarcinoma, surrounded by a prominent desmoplastic reaction. Hemorrhage and necrosis are also seen (asterisk). Hematoxylin and Eosin (HE). (b) Severe neutrophilic inflammation surrounding neoplastic tubular formations. Inset. Neoplastic cells showing positive immunoreaction against pan-keratin antibody. Mouse monoclonal anti- pankeratin (AE1/AE3/PCK26) antibody (c) Mesenteric tissue. Neoplastic cells infiltrating in the adjacent mesenteric tissue. HE. Inset. Mouse monoclonal anti- pankeratin (AE1/AE3/PCK26) immunolabelling (d) Mouse monoclonal anti- pankeratin (AE1/AE3/PCK26) immunolabelling

Histopathology showed disruption of 80% of the pancreatic parenchyma, which was invaded by a neoplastic growth of cuboidal to columnal cells, with tubular arrangement, with occasional cyst formation (Fig. 2a). The growth was clearly infiltrative, and tumor budding was seen in the periphery of the lesion. Mitotic figures were frequent (mitotic count 10 / 2.37 mm^2^). Multifocal areas of extensive hemorrhage and necrosis, along with a neutrophilic infiltrate were also reported (acute pancreatic necrosis) (Fig. 2b). A diagnosis of pancreatic ductal adenocarcinoma was made. Complete tumor-free margins were not achieved, as neoplastic cells were seen within the surrounding mesenteric tissue. Immunohistochemistry (IHC) evaluation confirmed pan-cytokeratin (DAKO Denmark) positivity (Fig. 2c and d). Therefore, according to the American Joint Committee on Cancer (AJCC) validated staging system, clinical stage IIA T3N0M0 was established (Pinard et al. 2021).

Adjuvant therapy with toceranib phosphate (Palladia® 2.4 mg/kg every Monday, Wednesday, and Friday) was commenced in combination with firocoxib (Previcox® 5 mg/kg every Tuesday, Thursday, and Saturday). Six weeks after starting toceranib, abdominal ultrasound did not identify signs of local recurrence or metastasis. Bloodwork did not reveal any abnormal parameters. After five months of treatment, the patient presented with grade 2 diarrhea (moderate limitation of activities of daily living), according to the criteria of Veterinary Cooperative Oncology Group—Common Terminology Criteria for Adverse Events (VCOG-CTCAEv2) (LeBlanc et al. 2021) which responded to gastrointestinal diet (Royal Canin®).

Treatment with toceranib and firocoxib was continued for a total of 6 months since diagnosis. Routine restaging was performed at seven months after diagnosis (one month after finishing toceranib phosphate), with abdominal ultrasound and thoracic radiographs that were performed and evaluated by a board-certified radiologist. No evidence of local recurrence or metastatic disease was found.

Eleven months after diagnosis, the patient presented for routine restaging. The owner described some lethargy, and abdominal pain was evident on palpation. A 13 mm mass in the liver parenchyma was reported on abdominal ultrasound. Ultrasound-guided fine needle aspirates were collected from the hepatic mass. Cytology revealed very abundant cellularity. In addition to clusters of hepatocytes with moderate to intense non-lipid vacuolar degeneration, there were abundant non-hepatic epithelial cells, distributed in clusters and acini, with a high nucleus/cytoplasm ratio, scant basophilic cytoplasm with vacuoles, pleomorphism and nuclear anisokaryosis. These findings were compatible with metastasis from the primary tumor. Therefore, the patient had a progression-free interval (PFI) of 305 days. Treatment with toceranib phosphate and firocoxib was restarted at the same previous dose.

The abdominal ultrasound performed two months after the re-commencement of toceranib phosphate showed that the mass had increased its size by 60% (20 mm). No clinical improvement was observed with the re-starting of toceranib phosphate and the patient developed anorexia, so treatment was discontinued. One month after the treatment was discontinued, the patient died of sudden death (445 days after diagnosis) despite a progressive deterioration with increasing anorexia, lethargy, and abdominal pain. The owners refused to allow the necropsy.

## Discussion

In the present case, surgical excision of a pancreatic carcinoma, combined with toceranib phosphate and firocoxib, achieved complete resolution of clinical signs and provided a survival time of 445 days. To our knowledge, this is the longest survival time reported in the literature for a dog diagnosed with exocrine pancreatic carcinoma; this tumor has a short survival time due to its aggressiveness and the high incidence of metastases at presentation (Pinard et al. 2021; Musser and Johannes 2021).

Canine pancreatic carcinoma is an aggressive uncommon cancer, with a high probability of metastasizing, which has been poorly described in veterinary literature in dogs (Aupperle-Lellbach et al. 2019; Pinard et al. 2021; Musser and Johannes 2021). The mean age at the time of diagnosis of pancreatic carcinomas is 9.5 years in dogs (Aupperle-Lellbach et al. 2019; Musser and Johannes 2021), being in this case 10-year-old at the time of diagnosis. The history and clinical signs of canine exocrine pancreatic adenocarcinoma are usually non-specific, such as; vomiting, regurgitations, anorexia and weight loss (Pinard et al. 2021; Musser and Johannes 2021). In the present case, clinical signs included anorexia, vomiting, abdominal pain and lethargy.

The diagnosis of pancreatic carcinomas is fundamentally based on abdominal ultrasound, and ultrasound-guided fine needle aspiration for cytology. Ultrasonography can indicate the presence of a large mass in the pancreas, but in some cases, the adenocarcinoma can mimic pancreatitis when there is no obvious mass (Bennett et al. 2001). Contrast-enhanced ultrasonography has been proposed as a possible method to establish different enhancement patterns between exocrine and endocrine pancreatic tumor. A study described adenocarcinomas showed hypoechoic and hypovascular lesions, whereas insulinomas showed uniformly hypervascular lesions (Vanderperren et al. 2014). In this case, a 6 cm x 4.1 × 3.5 cm solitary mass was detected in the cranial abdomen by an abdominal ultrasound. The utility of advanced imaging such as CT scan have not been extensively described for exocrine pancreatic tumors in veterinary literature (Pinard et al. 2021). However in this case, a CT scan was performed, both with diagnostic and surgical planning purposes. Blood count and serum biochemistry may also show non-specific findings such as slightly elevated liver enzymes and alkaline phosphatase (ALP) (Aupperle-Lellbach et al. 2019; Withrow et al. 2020; Musser and Johannes 2021). In a study of 22 canine patients with pancreatic carcinoma, some biochemical changes were described, such as elevations in canine pancreatic lipase immunoreactivity (CPLI), ester lipase (DGGR lipase), canine trypsin-like immunoreactivity (cTLI), C-reactive protein (CPR), alanine aminotransferase (ALT) and alkaline phosphatase (ALP) (Aupperle-Lellbach et al. 2019). However, in this case, biochemical parameters were within the reference interval.

The presence of pancreatitis has been described as a common histopathological finding in patients with pancreatic carcinoma (Aupperle-Lellbach et al. 2019). Differentiating between pancreatic carcinoma and pancreatitis based on clinical signs, serum biochemistry or abdominal ultrasound can be challenging (Withrow et al. 2020). Patients with pancreatitis may present with abdominal pain, vomiting, altered liver enzymes and ALP, and ultrasound imaging can be similar in cases of pancreatitis and diffuse pancreatic adenocarcinoma (Pinard et al. 2021). In this case, the combination of ultrasound and CT indicated neoplasia was more likely than other differential diagnoses, such as abscess, pancreatitis, or granuloma.

Although a definitive diagnosis can sometimes be made based on cytology alone (Bennett et al. 2001), biopsy sampling, whether it is though exploratory celiotomy or laparoscopy, is often necessary. In this case, cytology did not reveal neoplastic cells, but a neutrophilic inflammation. This can be explained by the secondary neutrophilic pancreatitis and pancreatic necrosis reported in the histological evaluation, which obscured the underlying neoplasm. In canine and feline patients, concurrent inflammation has been determined as a major obstacle in the utility of cytology for the diagnosis of pancreatic neoplasia (Withrow et al. 2020). In addition, although cytology has been determined to have a high diagnostic accuracy in the diagnosis of acinar adenocarcinomas, fine-needle aspirates in ductal adenocarcinomas do not usually achieve a definitive diagnosis (Bennett et al. 2001).

In human medicine, pancreatic ductal adenocarcinoma is a prevalent and highly malignant digestive system tumor that often requires surgical intervention for treatment. Pancreatic resection remains one of the most critical areas of gastrointestinal surgery and is associated with up to 50% morbidity and 8% mortality (Dominguez-Comesaña et al. 2013). Pancreaticoduodenectomy (PD) and total pancreatectomy (TP) are standard procedures for pancreatic head tumors, with PD involving resection of the pancreatic head, duodenum, part of the proximal jejunum, gallbladder, and distal stomach. PD is commonly used and is associated with a high recurrence rate due to the aggressive nature of the pancreatic ductal adenocarcinoma. TP, although sparing no part of the pancreas, has been introduced to improve surgical outcomes and minimize complications associated with reconstruction (Hong et al. 2023). Importantly, these surgical choices have inherent challenges, as pancreaticoduodenectomy is associated with significant morbidity (33–64%) and mortality (5–10%), underscoring the need for careful consideration of the anatomical and pathological aspects to optimize outcomes (Fernández-Cruz et al. 2008). Comparatively, in veterinary medicine, pancreatectomy is more frequently described as a surgical treatment in dogs, primarily due to the challenges and higher complication rates associated with pancreaticoduodenectomy (Wouters et al. 2011). The canine pancreas has a significant regenerative capacity that allows partial pancreatectomy, especially if the accessory pancreatic duct is preserved. This approach allows resection of a significant portion (75–90%) of the pancreas without compromising exocrine or endocrine function. However, if the accessory pancreatic duct is involved, pancreaticoduodenectomy is recommended (Cornell and Tobias 2018). The choice of partial pancreatectomy in our patient was substantiated by the caudal location of the tumor, which ensured a considerable distance from the pancreatic duct and the accessory pancreatic duct, accounting for potential anatomical variations. Pancreaticoduodenectomy was not considered, due to the apparent lack of duodenal involvement in the neoplasia. Besides, if the liver, peritoneal cavity or draining lymph nodes show signs of tumor positivity, it is generally advisable to avoid aggressive surgical procedures. This is because there is an increased risk of complications with no clear benefit in terms of survival or overall quality of life (Withrow et al. 2020).

At the time of initial presentation, no metastatic lesion was observed on abdominal ultrasound, nor on CT scan or during laparotomy. This is in contrast with previous studies, in which a high percentage of patients with pancreatic carcinoma had metastasis at the time of diagnosis (Pinard et al. 2021; Musser and Johannes 2021). A study in dogs reported that more than 78% of patients presented with overt metastatic disease at diagnosis (Pinard et al. 2021). In this case, the patient had a well-differentiated tumor, which is usually less aggressive. This could explain why the tumor was localised at the time of diagnosis and no metastases were found, making surgery a reasonable option. In human literature, the histopathological assessment is an essential prognostic factor (Silvanto et al. 2019).

The role of adjuvant therapy in pancreatic carcinomas remains controversial in people and dogs. To date, chemotherapy and radiation have shown no major benefit in human patients, although chemotherapy often improves the quality of life even if an increased survival is not achieved (Oettle et al. 2007). However, some studies have reported that, gemcitabine chemotherapy appears to have some efficacy, as well as tyrosine kinase inhibitor (TKIs) in humans (Gupta and El-Rayes 2008) and cats (Linderman et al. 2013; Todd and Nguyen 2020). Only one study has observed that toceranib may have biologic activity in dogs with pancreatic carcinoma (Musser and Johannes 2021).

Elevated expression of several tyrosine kinase receptors, such as the vascular endothelial growth factor receptor (VEGFR), has been found in human pancreatic carcinoma, and is associated with a worse clinical outcome (Gupta and El-Rayes 2008). Toceranib phosphate is a tyrosine kinase inhibitor (TKI) that blocks VEGFR, among other tyrosine kinase receptors (Musser and Johannes 2021). In humans, treatment of pancreatic neoplasia commonly relies on a pharmacological therapy, targeting VEFGFR among others (Gupta and El-Rayes 2008), which has some analogy to toceranib phosphate targeting tyrosine kinase receptors. Actually, other tyrosine kinase inhibitors, such as sunitinib, have demonstrated some efficacy in humans with pancreatic cancer (Hubner and Valle 2011). Nevertheless, a significant number of patients do not respond to this treatment, and mutations in the KRAS gene have been found to be one of the reasons behind this failure. KRAS mutations are commonly identified in human ductal carcinomas, whereas they are rarely reported in acinar carcinomas (Cancer Genome Atlas Research Network and Cancer Genome Atlas Research Network 2017), being the latter the most common subtype in dogs. Recent investigations in veterinary medicine have found that these mutations appear to be absent in canine pancreatic carcinomas (Crozier et al. 2016) but studies specifically regarding ductal carcinomas are lacking. The presence or absence of this mutation was not determined in the present case. However, given the apparent clinical benefit of toceranib phosphate in our patient, it could be hypothesized that mutations in KRAS gene in dogs are not as frequent as in human medicine.

In this case, toceranib phosphate was well tolerated and provided modest clinical benefit (partial response, or stable disease ≥ 10 weeks). These results are in line with those obtained in a small number of canine patients (Pinard et al. 2021; Musser and Johannes 2021), and in a larger number of feline patients (Todd and Nguyen 2020; Rosario et al. 2023), and in humans with exocrine pancreatic cancer (Thompson and Wood 2020).

In our case, toceranib phosphate was used in combination with firocoxib. This combination has not been described before in pancreatic carcinoma, but it is common as therapy for other tumors (Withrow et al. 2020).

Previous toceranib phosphate therapy described in dogs was administered at a mean dose of 2.5 mg/kg (range: 2.2 to 2.8 mg/kg) 3 days per week, with toceranib given as a single agent against macroscopic disease (Musser and Johannes 2021). However, in our case, toceranib was used as adjuvant therapy in combination with firocoxib, with a similar dose of toceranib phosphate (2.4 mg/kg 3 days per week) was administered. Although some side effects of toceranib administration have been described, such as mild anorexia, nausea, diarrhea and neutropenia in dogs, in our case only occasional diarrhea was observed.

As most patients with pancreatic adenocarcinoma have extensive disease at the time of diagnosis, and many are euthanised before or shortly after initiation of treatment, low survival rates have been reported, with one study in 23 dogs describing a median survival of only 1 day (Pinard et al. 2021). Other studies in canine adenocarcinoma patients treated with toceranib phosphate after surgery or without surgery reported a median overall survival of 89.5 days (range: 14–506 days) (Musser and Johannes 2021). A recent study in feline patients with pancreatic adenocarcinoma treated with toceranib phosphate, with or without surgical resection, reported a median survival of 97 days (range: 1 to 1666 days) (Rosario et al. 2023). Another single patient report describes that the cat survived more than 1436 days after treatment with surgical resection and toceranib phosphate (Todd and Nguyen 2020). This long survival time in our patient could be related to an early diagnosis of the tumor without metastatic disease at that time, the well-differentiated histopathologic characteristics, which suggest a less aggressive behavior of the tumor, and the potential effectiveness of a combined treatment of surgical resection and toceranib phosphate-firocoxib therapy. Nevertheless, being a single patient case report obviously limits the interpretation of treatment efficacy and extrapolation of survival times reported here.

In conclusion, the results of this case suggest that the combination of surgery with firocoxib and toceranib phosphate could be considered for the treatment of pancreatic adenocarcinoma to control the disease and prolong survival time in dogs. Future prospective studies evaluating the use of toceranib after surgical resection in dogs with pancreatic adenocarcinoma are needed.
